# Variability of Anthocyanin Concentrations, Total Metabolite Contents and Antioxidant Activities in Adzuki Bean Cultivars

**DOI:** 10.3390/antiox11061134

**Published:** 2022-06-09

**Authors:** Kebede Taye Desta, Hyemyeong Yoon, Myoung-Jae Shin, Sukyeung Lee, Xiao-Han Wang, Yu-Mi Choi, Jung-Yoon Yi

**Affiliations:** National Agrobiodiversity Center, National Institute of Agricultural Sciences, Rural Development Administration, Jeonju 54874, Korea; kehasiet20@rda.go.kr (K.T.D.); hyemyeung1@rda.go.kr (H.Y.); smj1204@rda.go.kr (M.-J.S.); reset00@korea.kr (S.L.); wang0530@rda.go.kr (X.-H.W.)

**Keywords:** adzuki bean, antioxidant activity, anthocyanin, seed coat, phenolic content, saponin content, *Vigna angularis*

## Abstract

In this study, adzuki bean cultivars including Arari, Chilbopat, Geomguseul, and Hongeon were recently cultivated, and the concentrations of seven individual anthocyanins were determined in their seed coats for the first time. Moreover, the variations of total saponin content (TSC), total phenolic content (TPC), 1,1-diphenyl-1-picrylhydrazyl (DPPH) radical scavenging activity, Trolox equivalent antioxidant capacity (TEAC), and ferric reducing antioxidant power (FRAP) between defatted and undefatted extracts of whole seeds, seed coats, and dehulled seeds of each were analyzed. The anthocyanins were detected only in the black seed-coated cultivars and delphinidin-3-*O*-glucoside was dominant in both Geomguseul (12.46 mg/g) and Chilbopat (10.88 mg/g) followed by delphinidin-3-*O*-galactoside. TSC and TPC were in the ranges of 16.20–944.78 mg DE/g and 0.80–57.35 mg GAE/g, respectively, and each decreased in the order of seed coats > whole seeds > dehulled seeds regardless of extract type. The antioxidant activities also showed similar patterns of variation. Geomguseul seed coats outweighed the remaining cultivars in terms of TPC and FRAP activity (*p* < 0.05). Generally, significant variations of metabolite contents and antioxidant activities were observed between cultivars and across their seed parts (*p* < 0.05). Thence, black seed-coated adzuki beans could be excellent sources of anthocyanins and antioxidants.

## 1. Introduction

Adzuki bean (*Vigna angularis* [Willd.] Ohwi and Ohashi), a legume plant, is widely cultivated in East Asia countries, including Japan, Korea, and China during the warm season. The plant has been used as a traditional medicine for a long time, and different adzuki bean seed products are consumed during the traditional festivals of these countries [1,2]. The seeds are also the major ingredients for preparing Chinese and Japanese sweets [3]. In Korea, adzuki bean seeds are locally named Pat, and they are mainly used for the preparations of soup, paste, and porridge [4]. In recent years, countries such as Australia and Canada, among others, also started producing and exporting adzuki bean signifying the potential of the legume to be a future commercial crop [5]. 

Previous studies verified that adzuki bean seeds are rich sources of both nutritional and non-nutritional metabolites. Compared with the non-nutritional metabolites, the nutritional components of adzuki beans have been widely investigated [6,7,8,9]. Carbohydrates are the most abundant nutritional components. Moreover, vitamins, fatty acids, minerals, protein, and fibers are also abundant in the seeds [8,9]. Among the non-nutritional secondary metabolites, saponins and polyphenolic compounds such as azukisaponins, flavonoids, proanthocyanidins, and anthocyanins are ubiquitous, the latter contributing to the pigmentation of their seed coats [1,10,11,12,13,14]. Due to the abundance of such wide classes of metabolites, adzuki beans are known to possess several health-promoting and disease-deterrence properties. Many in vivo and in vitro pharmacological investigations confirmed the antidiabetic, antiobesity, antioxidant, and anticancer properties of adzuki bean seeds [14,15]. Moreover, foods augmented with adzuki bean seeds were found to possess atypical colors that lift consumers’ preferences and show enhanced nutritional qualities and biological activities [3,16,17]. Owing to all these, researchers have been attempting to improve the quality and production of adzuki bean cultivars through breeding. Compared with other major crops such as rice, wheat, and barley, however, the breeding progress is far from satisfactory, signifying the need for multifaceted investigations [18,19,20].

Several factors including temperature, light, latitude, and precipitation affect the metabolite contents and nutritional qualities of adzuki bean seeds [20,21]. Moreover, the chemical compositions of adzuki bean seeds vary among different cultivars and according to their seed color, size, and shape [22,23,24,25]. Few studies also signified that the biological activities and distribution levels of different classes of metabolites could vary among the various seed parts. For instance, a recent investigation outlined the possible use of adzuki bean seed coats as preservatives for raw beef owing to the characteristic bacterial inhibitory effect of their polyphenols [26]. In another study, significant variations of metabolite contents and biological activities between the different seed parts of a Chinese adzuki bean cultivar were reported [14]. Moreover, other studies showed the dominance of anthocyanins and proanthocyanidins in the seed coats contributing to enhanced biological activities apart from their role in seed coat pigmentation [27,28]. In general, like in other crops, environmental, genetic, and crop management factors affect the metabolite contents in adzuki beans. Investigating the impacts of these factors on seeds’ chemical compositions could help to maximize the production of good-quality adzuki beans and increase their commercial values [29].

There are several commercialized adzuki bean cultivars in Korea: Arari, Chilbopat, Geomguseul, and Hongeon being the most popular [30]. Hongeon and Arari are red-seed-coated cultivars, whereas Geomguseul and Chilbopat are black-seed-coated cultivars. Additionally, previous studies investigated the metabolites contents, nutritional compositions, and biological activities of some Korean adzuki bean cultivars [4,30,31,32]. However, existing studies targeted a single cultivar and/or plant part. Moreover, the differences in metabolite contents and biological activities between Korean adzuki bean cultivars and their various plant and/or seed parts still need further investigation. This study aimed to determine the concentrations of seven individual anthocyanins including cyanidin-3-*O*-glucoside (C-3-*O*-G), delphinidin-3,5-*O*-diglucoside (D-3,5-*O*-di-G), delphinidin-3-*O*-galactoside (D-3-*O*-Ga), delphinidin-3-*O*-glucoside (D-3-*O*-G), delphinidin-3-*O*-rutinoside (D-3-*O*-Ru), petunidin-3-*O*-glucoside (Pt-3-*O*-G), and petunidin-3-*O*-galactoside (Pt-3-*O*-Ga) in the seed coats of recently cultivated four popular Korean adzuki bean cultivars (Arari, Chilbopat, Geomguseul, and Hongeon) for the first time. Furthermore, the relative total saponin content, total phenolic content, 1,1-diphenyl-1-picrylhydrazyl (DPPH) radical scavenging activity, Trolox equivalent antioxidant activity (TEAC), and ferric reducing antioxidant power (FRAP) of the defatted and undefatted seed coats, dehulled seeds, and whole seeds extracts were assessed. The results of this study could provide background knowledge regarding the variation in anthocyanin contents, total phenolic and total saponin contents, and antioxidant activities between adzuki bean cultivars and across their seed parts. Moreover, the study could initiate similar investigations of other adzuki bean genetic materials and promote their use in food industries. 

## 2. Materials and Methods

### 2.1. Chemicals and Reagents

Water was purchased from Thomas Scientific (Philadelphia, PA, USA), diosgenin from PhytoLab (Vestenbergsgreuth, Germany), sulfuric acid from DAEJUNG Chemicals (Siheung-si, Korea), and Pt-3-*O*-G from BioCrick BioTech (Chengdu, China). Methanol and ethanol were ordered from Fisher Scientific (Pittsburgh, PA, USA). The other chemicals and reagents including petroleum ether, anhydrous sodium carbonate (Na_2_CO_3_), vanillin, gallic acid, Folin–Ciocalteu phenol reagent, L-ascorbic acid, sodium carbonate, potassium ferricyanide, trichloroacetic acid, ferric chloride, 1,1-diphenyl-2-picrylhydrazyl (DPPH) radical, 2,2′-azino-bis(3-ethylbenzothiazoline-6-sulfonic acid) diammonium salt (ABTS), 6-hydroxy-2,5,7,8-tetramethylchroman-2-carboxylic acid (Trolox), C-3-*O*-G, D-3-,5-*O*-di-G, D-3-*O*-Ga, D-3-*O*-G, D-3-*O*-Ru, and Pt-3-*O*-Ga were obtained from Sigma Aldrich (St. Louis, MO, USA). All the chemicals and reagents were of analytical grade.

### 2.2. Seed Materials, Cultivation, and Sample Preparation 

**Figure 1:**
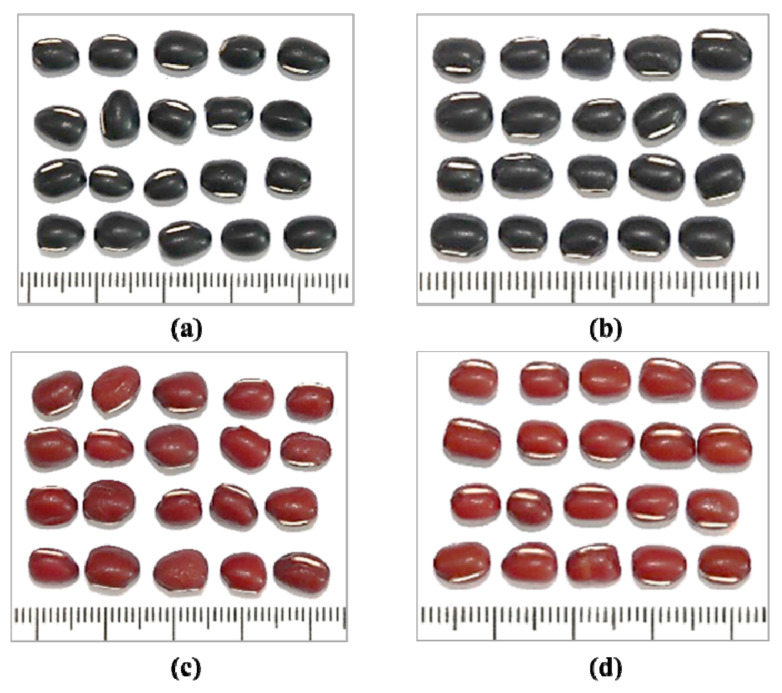
Seed samples of four Korean adzuki bean cultivars. (**a**): Chilbopat; (**b**): Geomguseul; (**c**): Arari; (**d**): Hongeon.

The seeds of the four Adzuki bean cultivars including Arari, Chilbopat, Geomguseul, and Hongeon (Figure 1) were obtained from the gene bank at the National Agrobiodiversity Center (Jeonju, Korea) and cultivated during the cropping season of the country on a clay loam soil experimental field found at the center (latitude/longitude: 30°49′38.37″ N/127°09′07.78″ E). 

In brief, two seeds in ten replicates were sown for each cultivar on 23 June 2020, at a spacing of 15 cm in 90 cm apart rows. The cultivation period lasted until late September of the same year. NPK fertilizer at a ratio of 3:3:3.4 kg a^−1^ was applied, and the growing conditions were maintained uniform for all the cultivars. The average temperature and precipitation were 23.4 °C and 187.5 mm in June, 23.5 °C and 644.7 mm in July, 27.2 °C and 471.5 mm in August, and 21.3 °C and 135.2 mm in September during the cultivation year, respectively. The agronomical features of the adzuki beans were inspected and recorded during the growth period. Matured beans were hand-harvested, dried in an LP500 freeze-dryer (ilShinBioBase, Dongducheon, Korea), and the seed coats (hull) were hand-peeled and separated the whole seed. Then, the seed parts (seed coat, whole seed, and dehulled seeds) were powdered using 3 mm stainless beads (TissueLyser II, Qiagen, Germantown, MD, USA), and stored at −20 °C in sealed plastic bags pending extraction. 

### 2.3. Extraction and Quantification of Anthocyanins 

The extraction of anthocyanins was conducted using the seed coat samples of each cultivar according to the method of Wu et al. [33] with some modifications. In brief, 50 mg of powdered seed coat sample of each cultivar, in triplicate, was mixed with 5 mL of a pre-chilled methanol solution containing 1% (*v*/*v*) of concentrated hydrochloric acid. The mixture was then sonicated in an ice bath for 45 min followed by centrifugation (3134× *g*) for 10 min. The supernatant was retained, and the extraction process was repeated one more time for the residue using 2.5 mL of the solvent. The combined supernatant was then passed through a 0.45 µm syringe filter into an injection vial and made ready for high-performance liquid chromatography (HPLC) analysis. The target anthocyanins including C-3-*O*-G, D-3,5-*O*-di-G, D-3-*O*-Ga, D-3-*O*-G, D-3-*O*-Ru, Pt-3-*O*-Ga, and Pt-3-*O*-G were identified and quantified using a reverse-phase 1260-Infinity Quaternary HPLC system (Agilent Technologies, Santa Clara, CA, USA) equipped with a diode-array-detector (DAD). An Inertsil ODS-3 column (250 × 4.6 mm, 5 μm; GL Sciences, Tokyo, Japan) was used for separation and maintained at 35 °C throughout the analysis. The mobile phase was a binary solvent system of water (A) and methanol (B) each containing 5% formic acid. The gradient condition started with 15% of solvent B followed by an increase to 18% at 10 min, to 20% at 25 min, to 21% at 28 min, to 30% at 30 min, and to 45% at 34 min. The final condition was maintained isocratic for one more minute and the total run time lasted for 35 min with an additional post-run of 5 min. The flow rate was 1 mL/min throughout the analysis, whereas the sample injection volume was 2 µL. The acquired chromatograms were read at λ_max_ 530 nm, and the retention times of the corresponding external standards were compared to confirm the identity of individual anthocyanins. For quantification, calibration curves (*R*^2^ > 0.999) were plotted from peak area responses of each standard at five concentration levels (100, 50, 25, 10, and 5 mg/L). The concentrations of individual anthocyanins were reported as mg/g of dried seed coat sample from triplicate measurements. 

### 2.4. Extract Preparation for Determination of Total Contents and Antioxidant Activities 

The preparation of defatted and undefatted extracts from each seed part was conducted according to previously reported methods with slight modifications [34]. In each case, 0.1 g of seed coat sample, and 0.2 g of whole seed and dehulled seed samples, each in triplicate, were used as described below.

#### 2.4.1. Preparation of Defatted Extracts

Initially, the measured amount of each sample (seed coat, whole seed, and dehulled seed) was separately mixed with 5 mL of petroleum ether, defatted via shaking on an automated orbital shaker (WITEG Labortechnik, Wertheim, Germany) for 4 h, and filtered. The residues were mixed with another 5 mL of the solvent and the defatting process was repeated one more time for 2 h. Then, each of the defatted residues was mixed with 2.5 mL of 80% methanol (aqueous), and extracted by sonication at 25 °C in the dark. After 45 min, the mixtures were taken off and centrifuged (3134× *g*) for 15 min using a high-speed centrifuge (Labogene, Daejeon, Korea). The upper supernatants were retained, and the extraction process was repeated one more time for each residue. The combined supernatants were then used for the determination of total saponin content, total phenolic content, DPPH-radical scavenging activity, Trolox equivalent antioxidant activity (TEAC), and ferric reducing antioxidant power (FRAP). All analyses were conducted within 72 h after sample extraction, and extracts were stored at −20 °C when not in use. 

#### 2.4.2. Preparation of Undefatted Extracts

To prepare the undefatted extracts, samples were directly mixed with 2.5 mL of 80% methanol and sonicated for 45 min at 25 °C in the dark. The extraction process was repeated one more time using the residue and 2.5 mL of the extraction solvent. Then, the mixtures were centrifuged each time (3134× *g*) as described above. For each sample, the upper supernatant was separately collected, combined, and used for metabolite contents and antioxidant activity analyses within 72 h after extraction. 

### 2.5. Determination of Total Saponin Content (TSC)

The TSC was determined spectrophotometrically according to a previously described method using a microplate reader with slight modification [35]. In brief, 25 µL of each of the defatted and undefatted sample extracts were separately mixed with an equal amount of freshly prepared 8% vanillin (*w/v* in ethanol) in a 96-well plate (Thermo Fisher Scientific, Roskilde, Denmark) and 250 µL of 72% sulfuric acid (*v/v* in water). The mixtures were incubated in a water bath at 60 °C for 10 min and subsequently cooled in an ice bath for 15 min. Then, the absorbance of each was measured at 544 nm on an Eon Microplate Spectrophotometer (Bio-Tek, Winooski, VT, USA). Several concentrations of diosgenin (0.10–2.00 mg/mL) were prepared and used to plot calibration curves (*R*^2^ > 0.999) and the TSC was then computed as milligram of diosgenin equivalent per gram of dried sample (mg DE/g).

### 2.6. Determination of Total Phenolic Content (TPC)

The TPC was estimated using the Folin–Ciocalteu colorimetric method following our recently reported protocol, with some modifications [36]. Briefly, 100 μL of each of the defatted and undefatted sample extracts were separately mixed with 100 μL of Folin–Ciocalteu reagent, and the mixtures were incubated at 25 °C in the dark. After 3 min, 100 μL of Na_2_CO_3_ solution (2%) was added, followed by a 30 min incubation in the dark. Then, the absorbance of each mixture was measured at 750 nm using an Eon Microplate Spectrophotometer (Bio-Tek, Winooski, VT, USA). Gallic acid was used as a standard (0.025–0.500 mg/mL) to plot calibration curves, and the TPC was computed as milligram of gallic acid equivalents per gram of dried sample (mg GAE/g).

### 2.7. Determination of Antioxidant Activities

Three in vitro assays were used to estimate the antioxidant activities of each seed part following our recently reported protocols with slight modifications, as described below [36]. During each assay, absorbance was measured using an Eon Microplate Spectrophotometer (Bio-Tek, Winooski, VT, USA).

#### 2.7.1. 1,1-Diphenyl-2-picrylhydrazyl (DPPH) Radical Scavenging Activity 

Initially, 100 μL of each of the defatted and undefatted sample extracts were separately mixed with an equal volume of freshly prepared DPPH solution (150 μM) in triplicate. Then, the mixtures were incubated for 30 min in the dark at 25 °C, and the absorbance of each was measured at 517 nm. A calibration curve was plotted using several concentrations (2.5–25.0 mg/L) of ascorbic acid (*R*^2^ > 0.999). The DPPH-radical scavenging activity was estimated as a milligram of ascorbic acid equivalent per gram of dried sample (mg AAE/g).

#### 2.7.2. Ferric Reducing Antioxidant Power (FRAP) Assay

During FRAP assay, 60 µL of each of the defatted and undefatted sample extracts were mixed with 150 µL of freshly prepared phosphate buffer (pH: 6.6, 0.2 M) and an equal volume of 1% potassium ferricyanide solution (K_3_Fe(CN)_6_) in triplicate. After 20 min of incubation at 50 °C, 150 µL of 10% trichloroacetic acid was added to each, followed by centrifugation (3134× *g*) for 10 min. Then, 100 µL of the supernatant was mixed with 100 µL of distilled water and 20 µL of 0.1% ferric chloride solution. The absorbance was measured at 700 nm after 10 min of incubation. Several concentrations of ascorbic acid (10–150 mg/L) were used to plot a calibration curve and FRAP values were expressed as mg AAE/g.

#### 2.7.3. Trolox Equivalent Antioxidant Capacity (TEAC)

Initially, 10 μL of each of the defatted and undefatted sample extracts were separately mixed with 150 μL of ABTS^•+^ working solution in triplicate. Then, the mixtures were incubated for 3 min at 25 °C in darkness, and the absorbance was measured at 734 nm. Trolox (10–250 mg/L) was used as a standard to plot calibration curves (*R*^2^ > 0.999) and the TEAC was determined as milligram of Trolox equivalent per gram of dried sample (mg TE/g).

### 2.8. Statistical Analysis

Results were expressed as mean ± standard deviation (SD) from triplicate measurements unless specified. Statistical analysis was conducted using analysis of variance (ANOVA) followed by Duncan’s multiple range test, and mean differences at *p* < 0.05 were considered significant. Total anthocyanin content was estimated as the sum of the levels of detected individual anthocyanins. Pearson correlation analysis was performed to view the association between metabolite contents and antioxidant capacities. All the other statistical analyses were conducted using xlstat software (Addinsoft, Long Island, NY, USA). 

## 3. Results and Discussion 

### 3.1. Characteristics of Adzuki Bean Cultivars 

In addition to differences in growing conditions, genotype differences among adzuki bean cultivars could cause variations in agronomical features during their growth. Such differences in the physical appearances and quantitative traits, in general, determine their preference in food industries and breeding [37]. In this study, the most common agronomical traits were recorded from the field and laboratory inspections (Table 1). The adzuki bean cultivars did not show a wide variation in their qualitative characteristics. All the cultivars developed green hypocotyledon and their growth habit was determinate. Moreover, their flower colors were yellow except for Chilbopat which displayed light-yellow flowers. The pod color was gray in all the adzuki bean cultivars. In contrast, differences were observed concerning quantitative agronomical traits. Hongeon was the earliest to flower and mature taking 32 and 59 days, whereas Chilbopat was the latest to flower and mature at 60 and 95 days, respectively. Arari and Geomguseul took equal days to flowering (49 days) and days to maturity (90 days). Early maturing legumes are greatly foreseen in food industries and hence, Hongeon could be a suitable candidate [26]. The average number of seeds per pod was comparable among the cultivars, whereas the average number of pods per plant was the highest in Geomguseul (*n* = 60.0) followed by Chilbopat (*n* = 47.7), Hongeon (*n* = 31.3), and Arari (*n* = 24.0) (*p* < 0.05). Moreover, the average one-hundred seeds weight (HSW) decreased in the order of Chilbopat (17.2 g) > Arari (14.3 g) > Hongeon (14.0 g) > Geomguseul (13.9 g) (*p* < 0.05). Based on their HSW, adzuki beans are classified as small (<12 g), medium (12–18 g), and large (>18 g) seeds [24]. Accordingly, all the cultivars provided medium-size seeds, and the finding agreed with a previous report [30]. 

### 3.2. Variations of Anthocyanin Contents 

**Figure 2:**
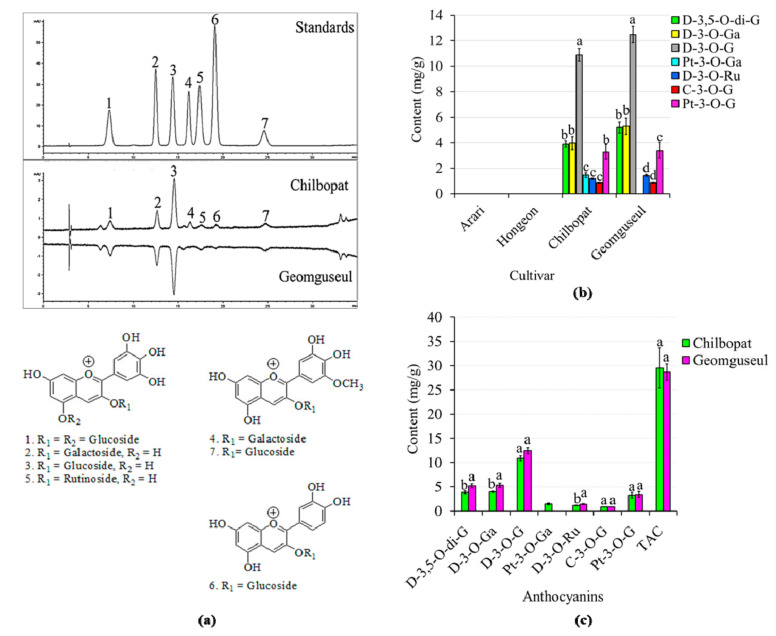
HPLC-chromatograms of standard anthocyanins, adzuki bean seed coat extracts, and structures of target anthocyanins (**a**); variation of anthocyanin contents within (**b**) and between (**c**) adzuki bean cultivars. Peak assignment and retention time (min): 1: D−3,5−*O*−di−G (7.34 min); 2: D−3−*O*-Ga (12.51 min); 3: D−3−*O*−G (14.40 min); 4: Pt−3−*O*−Ga (16.19 min); 5: D−3−*O*−Ru (17.40 min); 6: C−3−*O*−G (19.11 min); 7: Pt−3−*O*−G (24.61 min). Different letters on bars in a category indicate significantly different means (*p* < 0.05). C−3−*O*−G: cyanidin-3-*O*-glucoside; D−3,5−*O*−di−G: delphinidin-3,5-*O*-diglucoside; D−3−*O*-Ga: delphinidin-3-*O*-galactoside; D−3−*O*−G: delphinidin-3-*O*-glucoside; D−3−*O*−Ru: delphinidin-3-*O*-rutinoside; Pt−3−*O*−Ga: petunidin-3-*O*-galactoside; Pt−3−*O*−G: petunidin-3-*O*-glucoside; TAC: total anthocyanin content; t_R_: retention time.

Anthocyanins are highly concentrated in the inner palisade layer of legume seeds and play a vital role in seed pigmentation [38]. Compared with other legumes such as soybean, cowpea, lentil, and kidney bean, among others, the compositions and contents of anthocyanins in adzuki beans are hardly explored [39,40]. Previously, our colleagues at RDA (Jeonju, Republic of Korea) identified ten anthocyanins from the seed coats of one of the cultivars, Geomguseul, using NMR and LC-MS/MS techniques. In that study, however, the levels of the identified anthocyanins were left unquantified [4]. In this study, we targeted those anthocyanins with available standards including C-3-*O*-G, D-3,5-*O*-di-G, D-3-*O*-Ga, D-3-*O*-G, D-3-*O*-Ru, Pt-3-*O*-G, and Pt-3-*O*-Ga and studied their distribution and concentration in each of the four adzuki bean cultivars for the first time. The target anthocyanins were quantified using the corresponding external standards as described before. Figure 2a shows the representative HPLC-chromatograms of the standards and the adzuki bean samples. Our results found variations in both the distribution and concentration of the anthocyanins. All the seven individual anthocyanins were not detected in Hongeon and Arari, the two red-seed-coated adzuki beans. In contrast, all the anthocyanins except for Pt-3-*O*-Ga were detected in both the black-seed-coated adzuki beans including Chilbopat and Geomguseul. Pt-3-*O*-Ga was not detected in Geomguseul (Figure 2a,b). As outlined by Li et al. [37] and Chu et al. [41], such differences in the distribution of anthocyanins between colored adzuki beans could be due to the variation in the gene composition and enzymes responsible for anthocyanin biosynthesis. In terms of concentration, the dominance of D-3-*O*-G was observed in both Chilbopat (10.88 mg/g) and Geomguseul (12.46 mg/g) followed by D-3-*O*-Ga (3.97 and 5.30 mg/g, respectively) and D-3,5-*O*-di-G (3.89 and 5.21 mg/g, respectively) (*p* < 0.05). We also compared the relative concentrations of the commonly detected anthocyanins between the two black seed-coated cultivars. The levels of D-3,5-*O*-di-G, D-3-*O*-Ga, D-3-*O*-G, and D-3-*O*-Ru were higher in Geomguseul than in Chilbopat, each except for D-3-*O*-G, being significantly different (*p* < 0.05) (Figure 2c, Table S1). The concentrations of the remaining anthocyanins including C-3-*O*-G and Pt-3-*O*-G did not show significant variations between the two cultivars. The variation in total anthocyanin content, determined as the sum of individual anthocyanins, was also not significantly different (*p* < 0.05). The influence of seed coat color on the levels of anthocyanins has been widely investigated in other legumes [42,43,44]. Although adzuki beans are found in several seed coat colors, such studies are still scarce. Only recently, Zhao et al. [45] evaluated the relative abundance of anthocyanins in nine Chinese adzuki beans of different seed coat colors and found a high accumulation of anthocyanins in black seed coat adzuki beans. In another aspect, Yoshida et al. [46] compared the distributions and contents of anthocyanins in Japanese and Chinese adzuki beans with other legumes. These observations further signify the effects of genotype difference, origin, and cultivation year and place on the distribution and level of anthocyanins in adzuki bean genetic materials. Overall, our results assert the influence of seed coat color and genotype on the distribution and concentrations of individual anthocyanins in adzuki beans. Accordingly, the black seed coat adzuki bean cultivars including Chilbopat and Geomguseul could be ideal sources of anthocyanins. 

### 3.3. TSC, TPC, and Antioxidant Activities

In addition to anthocyanins, phenolic compounds and saponin components of adzuki beans collectively contribute to their health-promoting and disease deterrence properties and are receiving much attention in recent years [31,32,47,48]. In addition, the abundance of phenolic compounds could improve the quality and shelf life of other food products and hence, promote the use of adzuki bean seeds as natural antioxidants [26]. In this study, we investigated the levels of TSC and TPC of the four Korean adzuki bean cultivars and analyzed their distribution pattern in defatted and undefatted extracts of their whole seeds, seed coats, and dehulled seeds along with antioxidant activities. As shown in Table 2, TSC was in the ranges of 32.44–46.85, 629.20–842.63, and 16.79–25.11 mg DE/g in the defatted extracts and 33.86–45.44, 663.13–944.78, and 16.20–29.19 mg DE/g in the undefatted extracts of whole seeds, seed coats, and dehulled seeds, respectively. Likewise, the TPC level in the defatted extracts was in the ranges of 1.82–2.82 mg GAE/g (whole seeds), 39.70–55.08 mg GAE/g (seed coats), and 0.80–1.19 mg GAE/g (dehulled seeds). In the undefatted extracts, the TPC was in the ranges of 2.41–2.98, 39.48–57.35, and 0.99–1.31 mg GAE/g in the whole seeds, seed coats, and dehulled seeds, respectively. Studies that investigate the levels of TPC and TSC in the various seed parts of adzuki beans are scarce as described before. In general, the TPC levels observed in the whole seed samples fall within previously reported ranges [21,49]. The various antioxidant assays involve different reaction mechanisms, and hence, it is recommended to examine different assays to achieve a broader antioxidant activity assessment of plant and food extracts [36]. Here, we analyzed the antioxidant activities of each extract and seed part using three in vitro methods, including DPPH-radical scavenging activity, FRAP, and TEAC. The DPPH-radical scavenging activity was in the ranges of 3.08–3.98, 35.93–70.50, and 0.23–0.48 mg AAE/g in the defatted extracts, and 3.05–3.67, 35.26–71.07, and 0.24–0.51 mg AAE/g in the undefatted extracts of whole seeds, seed coats, and dehulled seeds, respectively, whereas FRAP was in the ranges of 2.77–4.88, 92.55–128.63, and 0.22–1.06 mg AAE/g in the defatted extracts, and 2.77–4.55, 89.25–135.30, and 0.20–1.07 mg AAE/g in the undefatted extracts of whole seeds, seed coats, and dehulled seeds, respectively (Table 3). Similarly, the TEAC was in the ranges of 7.21–13.72, 93.46–114.91, and 1.97–2.51 mg TE/g in the defatted extracts, and 8.18–13.77, 98.55–108.81, and 1.98–2.62 mg TE/g in the undefatted extracts of whole seeds, seed coats, and dehulled seeds, respectively. Previous studies also analyzed the antioxidant activities of adzuki bean extracts and reported wide-ranging results [21,24,49]. However, there are differences in extraction protocol, assays, and data reporting that make the comparison challenging. Compared with our result, a wider range but much lower ABTS activities (1.64–8.15 mg TE/g) were reported for the whole seed extracts of Korean adzuki beans and the difference in genotype and cultivation year could cause such variations [49]. In the following sections, the variations of TPC, TSC, and antioxidant activities in relation to extract type, seed parts, and cultivars are sequentially described.

#### 3.3.1. Effect of Defatting on TSC, TPC, and Antioxidant Activities

**Figure 3:**
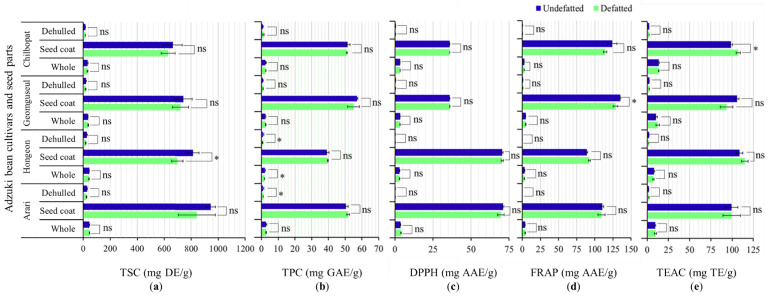
Variation of total saponin content (TSC) (**a**), total phenolic content (TPC) (**b**), DPPH-radical scavenging activity (**c**), Ferric reducing antioxidant power (FRAP) (**d**), and Trolox equivalent antioxidant activity (TEAC) (**e**) between defatted and undefatted extracts of seed coats, whole seeds, and dehulled seeds of four adzuki bean cultivars. * *p* < 0.05; ns Not significant.

The interaction of biomolecules with organic solvents during the defatting process could affect their extractability in aqueous solvents and could also lead to the loss of lipophilic secondary components. Moreover, it is one of the time-consuming steps during the extraction of natural antioxidants [50,51]. In this study, we initially investigated the influence of defatting on the levels of TSC and TPC in a specific seed part of the same cultivar along with antioxidant activities. Figure 3 shows the variations of TSC, TPC, and antioxidant activities between the defatted and undefatted extracts of each seed part in a cultivar. The numerical values can be read in Table 2 and Table 3. 

As shown in Figure 3a, the defatting process seemed to reduce the level of TSC in the seed coats and whole seeds of all the cultivars. However, the effect of defatting on the level of TSC in every seed part of all the cultivars was statistically insignificant (*p* < 0.05) except for Hongeon seed coats. The defatting step also reduced the level of TPC in the seed parts of all the adzuki bean cultivars except for the dehulled seeds of Chilbopat and Geomguseul, and the seed coats of Arari and Hongeon (Figure 3b). Once again, only in the dehulled seeds of Hongeon and Arari and the whole seeds of Hongeon that the defatting step conveyed significant variation in the level of TPC (*p* < 0.05). Concerning antioxidant activities, all the cultivars showed comparable DPPH-radical scavenging activities in both the defatted and undefatted extracts of their respective seed parts and no significant variation was observed (Figure 3c). By comparison, the undefatted seed coat extracts of Chilbopat and Geomguseul showed a relatively higher FRAP than the defatted seed coat extracts (Figure 3d). Contrary to this, the defatted seed coat extracts of Hongeon and Chilbopat showed a higher TEAC than the undefatted extracts (Figure 3e). Despite such differences, however, only the seed coats of Geomguseul in FRAP and Chilbopat in TEAC showed significant variations (*p* < 0.05) between the defatted and the undefatted extracts (Figure 3). The interaction of extract type (E) (defatted vs. undefatted) with cultivar (C) and seed part (S) was also investigated (Table 4). The influences of cultivar-extract type (C × E) interaction were insignificant in all the measured parameters. Moreover, the interaction of extract type and seed part (E × S) showed a significant effect on TSC and FRAP (*p* ≤ 0.01) but not on the other parameters. In recent years, several studies reported the effect of defatting on the concentrations of various nutritional components in commercial crops [51,52,53]. In contrast, the effect of defatting on the levels of non-nutritional components such as phenolics and saponins was hardly investigated, and variable results were reported where recorded. For instance, Mondor et al. [54] investigated the impact of defatting on TPC in the flour of two chickpea varieties and found no significant variation. In other studies, Buitimea-Cantua et al. [55] found a significant reduction in the level of TPC following defatting of red and white sorghums, whereas Nepote et al. [56] observed the opposite trend in peanuts. Specific to adzuki beans, studies showing the effect of defatting on the levels of TPC and TSC in the seed extracts, and their antioxidant activities have not been reported. Taking the observed exceptions into account, it can be suggested that sonication of the whole seeds of adzuki bean cultivars could provide saponin-rich extracts without performing an additional defatting step, at least when using 80% methanol as solvent. Likewise, direct sonication of the seed coats of adzuki bean cultivars could provide phenolic-rich extracts.

#### 3.3.2. Variations of TSC, TPC, and Antioxidant Activities across Seed Parts 

As previously outlined, several studies showed the variation in the distribution of different classes of bioactive metabolites between the different legume seed parts [48,57,58,59,60]. Taking these into account, the relative TSC and TPC levels between the seed coats, whole seeds, and dehulled seeds of each adzuki bean cultivar were analyzed. Likewise, the variations of each of the antioxidant activities were investigated. As shown in Table 2, the levels of both TSC and TPC significantly varied between the different seed parts (*p* < 0.05). Equally, the antioxidant activities including DPPH-radical scavenging activity, FRAP, and TEAC also significantly varied between the different seed parts (Table 3, *p* < 0.05). Specifically, the seed coats of all the adzuki bean cultivars contained a significantly high level of TSC reaching up to 944.78 mg DE/g (*p* < 0.05). In contrast, the dehulled seeds of all the adzuki bean cultivars provided the lowest TSC level. Similar trends were observed concerning TPC level and all the antioxidant activities each decreasing in the order of seed coats > whole seeds > dehulled seeds for all the cultivars regardless of extract type (Table 2 and Table 3). A previous study conducted using a Chinese adzuki bean cultivar also found higher levels of TPC and TSC, and pronounced antioxidant activities in the seed coats which agreed with our observations [15]. Compared with the dehulled seeds, the TPC level in the seed coats was 38.84 to 57.69-fold higher in all the cultivars (*p* < 0.05). Moreover, the seed coats showed >70-fold higher DPPH-radical scavenging activity than the dehulled seeds. Similarly, the FRAP and TEAC activities of the seed coats were >120 and >37-fold higher than the dehulled seeds, respectively. The distinct antioxidant activities of the seed coats could be associated with their high levels of TPC and TSC as previously noted in other legumes [57,58,60]. Moreover, the greater TSC, TPC and antioxidant activities observed in the whole seeds compared with the dehulled seeds in each cultivar indicate the contribution of the seed coats to the metabolite contents and antioxidant activities of the entire seeds. Interestingly, the interaction of seed part and cultivar (C × S) showed a significant effect on metabolites contents (TPC and TSC) as well as on all antioxidant activities (*p* ≤ 0.0001) (Table 4). Generally, our findings suggest that the seed coats of adzuki beans could be ideal sources of natural antioxidants owing to their greater concentrations of saponin and phenolic components.

#### 3.3.3. Variations of TSC, TPC, and Antioxidant Activities between Cultivars

Several studies showed that the difference in genetic composition causes variation in the concentration of various classes of metabolites between legume cultivars [19,22,24]. In this study also, significant variations of TSC and TPC were observed between the four adzuki bean cultivars for a similar seed part. Arari cultivar contained the highest level of TSC in all its seed parts except for its defatted whole seeds, whereas Chilbopat contained the lowest TSC levels in all its seed parts irrespective of extract type (Table 2). The TSC level in the seed coats of Arari cultivar differed significantly from that of Chilbopat, whereas the TSC level in the whole seeds and dehulled seeds differed significantly from those of Geomguseul and Chilbopat, regardless of extract type (*p* < 0.05, Table 1). These findings further signify the effect of seed coat color on the level of TSC. Likewise, the seed coats of Geomguseul had a significantly high level of TPC whereas Hongeon seed coats had the lowest TPC level regardless of extract type (*p* < 0.05). Furthermore, Arari whole seeds had the highest TPC in both extracts whereas Hongeon whole seeds had the lowest. In a previous study, TPC levels of 1.52 mg and 2.03 mg GAE/g were reported in the whole seeds of Arari and Geomguseul, respectively each being lower than the TPC levels found in this study for similar cultivars [49]. The same study reported a higher TPC level (2.69 mg GAE/g) in the whole seeds of Hongeon compared with our study (1.82 and 2.41 mg GAE/g for the defatted and undefatted extracts, respectively). The difference in extraction protocol, cultivation year and location could have brought the observed inconsistencies [21,22]. As stated before, the interaction of the seed part with the cultivar (C × S) had a significant effect on all measured parameters (Table 4). Moreover, the interaction of the three traits (C × E × S) had a significant effect on TEAC and FRAP alone (*p* ≤ 0.001). 

To the best of our knowledge, only Luo et al. [15] investigated the variations of TSC, TPC, and antioxidant activities between the seed coats, whole seeds, and dehulled seeds using a single Chinese adzuki bean cultivar named Jingnong #5. In that study, TPC levels of 54.45 mg GAE/g in the seed coats, 0.40 mg GAE/g in the dehulled seeds, and 7.63 mg GAE/g in whole seeds of the cultivar were reported. The TPC value found in the seed coats of this Chinese cultivar was close to those of Chilbopat, Geomguseul, and Arari seed coats found in our study, but much higher than the TPC level observed in Hongeon seed coats (39.70 mg GAE/g for defatted and 39.48 mg GAE/g for undefatted) (Table 2). In contrast, the TPC level in the dehulled seeds was much lower and that of whole seeds was much higher than all the cultivars considered in our study for the same seed parts. In another study, compared with all the cultivars in our study, a lower TPC (~0.81 mg GAE/g) was reported in the whole seeds of a Brazilian adzuki bean cultivar [61]. These observations denote the wide-ranging nature of the concentrations of metabolites in the seeds of adzuki bean cultivars. Once again, differences in genotype, place of cultivation, growing conditions, and extraction protocols could cause such discrepancies [62,63].

Regarding antioxidant activities, the seed coats of Geomguseul, whole seeds of Arari, and whole seeds of Hongeon showed the greatest DPPH-radical scavenging activity, FRAP, and TEAC, respectively compared with the other cultivars for the same seed part regardless of extract type (Table 3) (*p* < 0.05). Compared with our findings, a lower TEAC was reported for the whole seeds of Arari, Geomguseul, and Hongeon in a previous study [49], and the difference in extraction protocol and year of cultivation could be the cause of these differences. As stated before, the interaction of cultivar with seed part showed a significant effect on both metabolites’ contents and antioxidant activities. Moreover, the interaction of cultivar, extract type, and seed parts (cultivar × extract type × seed parts) had a significant effect on ABTS and FRAP but not on TPC, TSC and DPPH (Table 4). In general, our results showed the variation of metabolite contents and antioxidant activities between adzuki bean cultivars. Based on our results, it can be suggested that the Arari whole seeds could be excellent sources of TPC and TSC compared with the remaining cultivars. Geomguseul seed coats could also be ideal sources of antioxidants owing to their high level of TPC.

### 3.4. Principal Component and Correlation Analyses 

**Figure 4:**
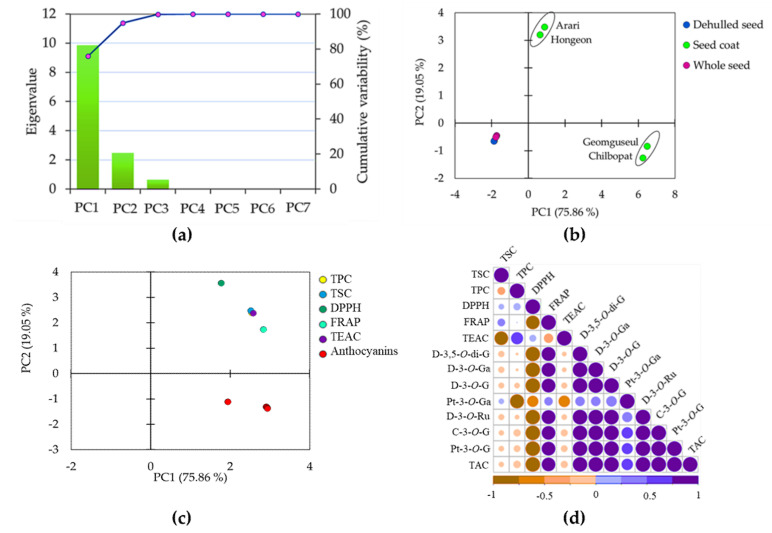
Scree plot of principal components (**a**), score plot of cultivars (**b**) and loading plot of variables (**c**) obtained from principal component analysis and pair-wise Pearson’s correlation matrix (**d**). C−3−*O*−G: cyanidin-3-*O*-glucoside; D−3,5−*O*−di−G: delphinidin-3,5-*O*-diglucoside; D−3−*O*-Ga: delphinidin-3-*O*-galactoside; D−3−*O*−G: delphinidin-3-*O*-glucoside; D−3−*O*−Ru: delphinidin-3-*O*-rutinoside; DPPH: DPPH-radical scavenging activity; FRAP: ferric reducing antioxidant power; Pt−3−*O*−Ga: petunidin-3-*O*-galactoside; Pt−3−*O*−G: petunidin-3-*O*-glucoside; TAC: total anthocyanin content; TEAC: Trolox equivalent antioxidant activity; TPC: total phenolic content; TSC: total saponin content.

Principal component (PCA) and Pearson’s correlation analyses were computed to further view the variations between the adzuki bean cultivars and their association to metabolite contents and antioxidant activities. All data obtained from the analysis of undefatted seed sample extracts including anthocyanin contents, TSC, TPC, and antioxidant activities were used, and some important correlations were observed (Figure 4). In the PCA, the first two principal components (PC1 and PC2) had eigenvalues of >1 and together explained 94.91% of the total variance (Figure 4a). Specifically, PC1 explained 75.86% of the variance whereas PC2 explained 19.05% of the total variance. As can be seen in the score plot, the seed coats were separated from the other seed parts and closely clustered according to their seed coat color (Figure 4b). 

The anthocyanins were the principal factors along PC1 and closely related to the black-seed-coated cultivars. Likewise, the TPC, TSC, TEAC, and DPPH were the major factors that explained the variation along PC2. FRAP showed comparable contributions along the axis of both principal components (Figure 4c). The overall observation further demonstrates the variations in metabolite contents and antioxidant activities between the adzuki bean cultivars and their seed parts. The Pearson’s correlation analysis also showed some important associations that support the PCA observation (Figure 4d). Both TPC and TSC were positively correlated to DPPH-radical scavenging activity although each association was not significant (*p* < 0.05). Likewise, TPC showed a strong and positive correlation with TEAC (*r* = 0.667) whereas TSC showed a weaker but positive correlation with FRAP activity (*r* = 0.253). As outlined before, the different degrees of association observed between TPC, TSC and antioxidant activities could be attributed to the variation in the mechanism of actions of these three assays [36]. Overall, our observations infer the contributions of both saponins and phenolics to the antioxidant properties of adzuki bean seeds [13,14,15]. Several studies investigated the association of TSC and TPC with antioxidant capacities in legumes. As observed in our results, some studies found the positive associations of TPC and TSC with antioxidant capacities whereas others indicated negative or weak associations [37,48,57,64]. It was interesting to note the anthocyanins showed a strong association with each other but showed an inverse association with both TSC and TPC which could be due to the difference in their biosynthetic pathways. Despite these, however, all the anthocyanins, except for Pt-3-*O*-Ga, showed a strong and positive correlation to FRAP (*r* ≥ 0.865) demonstrating their contribution to the antioxidant properties of the adzuki beans. Except for Pt-3-*O*-Ga, the least abundant anthocyanin, the remaining anthocyanins showed a significant and positive correlation with each other (*r* ≥ 0.984, *p* < 0.05).

## 4. Conclusions

In this study, four Korean adzuki bean cultivars were cultivated and the distribution and concentrations of seven anthocyanins in the seed coats of each were analyzed. Furthermore, the total phenolic content, total saponin content, DPPH-radicals scavenging activity, FRAP, and TEAC of their whole seeds, seed coats, and dehulled seeds were compared using defatted and undefatted extracts. Our findings revealed the variation in the distribution and levels of the anthocyanins between the cultivars. The black seed-coated cultivars were rich in anthocyanins and D-3-*O*-G was the most abundant metabolite. The influences of extract type, cultivar, seed parts, and their interactions on both total phenolic content, total saponin content, and antioxidant activities were also investigated. Among the seed parts, the seed coats of all the cultivars contained the highest levels of total phenolic and saponin contents. Moreover, the seed coats showed pronounced antioxidant capacities compared with the whole seeds and dehulled seeds in each cultivar. The metabolite contents and antioxidant activities also significantly varied between the cultivars. The interaction of cultivar and seed part was found to be an important parameter since it significantly affected all the measured parameters. In general, the seed coats of adzuki bean cultivars could be excellent sources of antioxidants for consumption and industrial use. Moreover, the high level of phenolic and saponin contents in the seed coats could contribute to an increased antioxidant capacity for the entire seed besides its role as a protective barrier for cotyledon. The results of this study could lay foundations for future investigations since previous studies dealing with anthocyanin contents and the various antioxidant extracts of adzuki bean seeds are infrequent. Moreover, further studies elucidating the main saponins and phenolic compounds responsible for the antioxidant activities of the seed coats and whole seeds of adzuki bean cultivars would be of interest.

## Figures and Tables

**Table 1:** Key agronomical characteristics of four adzuki bean cultivars cultivated in Korea.

Characteristic	Adzuki Bean Cultivar			
	Chilbopat	Geomguseul	Hongeon	Arari
	Qualitative			
Hypocotyledon color	Green	Green	Green	Green
Growth type	Determinate	Determinate	Determinate	Determinate
Flower color	Light-yellow	Yellow	Yellow	Yellow
Pod color	Gray	Gray	Gray	Gray
	Quantitative			
DF (days)	60	49	32	49
DM (days)	95	90	59	90
PPP (number, *n* = 3)	47.7 ± 16.4 ^ab^	61.0 ± 6.5 ^a^	31.3 ± 10.1 ^b^	24.0 ± 10.4 ^b^
SPP (number, *n* = 10)	6.1 ± 0.7 ^c^	6.9 ± 0.7 ^b^	6.6 ± 0.5 ^bc^	7.9 ± 0.7 ^a^
HSW (g, *n* = 3)	17.2 ± 0.1 ^a^	13.1 ± 0.0 ^d^	14.0 ± 0.1 ^c^	14.3 ± 0.1 ^b^

Different letters (^a–d^) in a row show significantly different means. DF: Days to flowering; DM: Days to maturity; HSW: One-hundred seeds weight; PPP: Pods per plant; SPP: Seeds per pod.

**Table 2:** Total saponin and total phenolic contents in the whole seeds, seed coats, and dehulled seeds of four Korean adzuki bean cultivars.

Metabolite Content	Extract	Seed Parts	Adzuki Bean Cultivars				Range	CV (%)
			Chilbopat	Geomguseul	Hongeon	Arari		
TSC (mg DE/g)	Defatted	Whole seed	32.44 ± 2.50 ^b,z^	37.93 ± 2.03 ^b,y^	42.38 ± 3.61 ^b,xy^	46.85 ± 0.55 ^b,x^	32.44–46.85	13.38
		Seed coat	629.20 ± 51.81 ^a,y^	721.12 ± 59.22 ^a,xy^	698.01 ± 41.90 ^a,xy^	842.63 ± 136.74 ^a,x^	629.20–842.63	10.66
		Dehulled seed	16.79 ± 0.74 ^b,y^	19.00 ± 3.09 ^b,y^	19.74 ± 1.78 ^b,y^	25.11 ± 2.74 ^b,x^	16.79–25.11	15.16
	Undefatted	Whole seed	33.86 ± 3.24 ^b,y^	36.92 ± 1.16 ^b,y^	45.44 ± 0.43 ^b,x^	45.12 ± 1.94 ^b,x^	33.86–45.44	12.56
		Seed coat	663.13 ± 70.47 ^a,z^	741.74 ± 66.66 ^a,yz^	814.20 ± 44.08 ^a,y^	944.78 ± 34.18 ^a,x^	663.13–944.78	13.10
		Dehulled seed	16.20 ± 1.81 ^b,y^	20.27 ± 2.95 ^b,y^	26.50 ± 4.13 ^b,x^	29.12 ± 1.58 ^b,x^	16.20–29.19	22.09
TPC (mg GAE/g)	Defatted	Whole seed	2.58 ± 0.07 ^b,x^	2.51 ± 0.15 ^b,x^	1.82 ± 0.13 ^b,y^	2.82 ± 0.29 ^b,x^	1.82–2.82	15.34
		Seed coat	51.01 ± 0.33 ^a,y^	55.08 ± 3.53 ^a,x^	39.70 ± 0.24 ^a,z^	51.82 ± 0.86 ^a,xy^	39.70–55.08	11.75
		Dehulled seed	1.19 ± 0.40 ^c,x^	1.05 ± 0.06 ^c,x^	0.80 ± 0.15 ^c,x^	1.07 ± 0.02 ^c,x^	0.80–1.19	14.12
	Undefatted	Whole seed	2.72 ± 0.07 ^b,xy^	2.66 ± 0.15 ^b,yz^	2.41 ± 0.13 ^b,z^	2.98 ± 0.29 ^b,x^	2.41–2.98	7.53
		Seed coat	51.85 ± 0.33 ^a,y^	57.35 ± 3.53 ^a,x^	39.48 ± 0.24 ^a,z^	50.85 ± 0.86 ^a,y^	39.48–57.35	13.02
		Dehulled seed	1.01 ± 0.40 ^c,y^	0.99 ± 0.06 ^b,y^	1.26 ± 0.15 ^c,x^	1.31 ± 0.02 ^c,x^	0.99–1.31	12.49

Different superscript letters (^a–c^) within a column of the same cultivar and extract denote statistically significant differences (*p* < 0.05). Different superscript letters (^x,y,z^) within a row and the same seed part denote statistically significant differences (*p* < 0.05). CV: Coefficient of variation; TPC: Total phenolic content; TSC: Total saponin content.

**Table 3:** Antioxidant activities of the whole seeds, seed coats, and dehulled seeds of four Korean adzuki bean cultivars.

Activity	Extracts	Seed Parts	Adzuki Bean Cultivars				Range	CV (%)
			Chilbopat	Geomguseul	Hongeon	Arari		
DPPH (mg AAE/g)	Defatted	Whole seed	3.43 ± 0.02 ^b,x^	3.33 ± 0.07 ^b,xy^	3.08 ± 0.23 ^b,y^	3.98 ± 0.20 ^b,w^	3.08–3.98	9.46
		Seed coat	35.93 ± 0.00 ^a,x^	36.09 ± 0.06 ^a,x^	70.50 ± 0.74 ^a,w^	69.61 ± 2.18 ^a,w^	35.93–70.50	32.10
		Dehulled seed	0.48 ± 0.05 ^c,w^	0.45 ± 0.06 ^c,w^	0.23 ± 0.05 ^c,x^	0.26 ± 0.01 ^c,x^	0.23–0.48	31.26
	Undefatted	Whole seed	3.41 ± 0.01 ^b,x^	3.37 ± 0.01 ^b,x^	3.05 ± 1.04 ^b,y^	3.67 ± 2.00 ^b,w^	3.05–3.67	6.48
		Seed coat	35.26 ± 0.26 ^a,x^	36.05 ± 0.06 ^a,x^	70.52 ± 4.07 ^a,w^	71.07 ± 5.05 ^a,w^	35.26–71.07	32.63
		Dehulled seed	0.48 ± 0.01 ^c,w^	0.51 ± 0.04 ^c,w^	0.31 ± 0.00 ^c,x^	0.24 ± 0.03 ^c,y^	0.24–0.51	29.38
FRAP (mg AAE/g)	Defatted	Whole seed	2.77 ± 0.21 ^b,y^	4.88 ± 0.37 ^b,w^	2.86 ± 0.28 ^b,y^	3.97 ± 0.26 ^b,x^	2.77–4.88	22.36
		Seed coat	114.28 ± 2.31 ^a,x^	128.63 ± 3.23 ^a,w^	92.55 ± 1.30 ^a,y^	109.23 ± 4.85 ^a,x^	92.55–128.63	11.60
		Dehulled seed	0.46 ± 0.03 ^b,x^	1.06 ± 0.01 ^c,w^	0.22 ± 0.06 ^c,y^	0.25 ± 0.04 ^b,y^	0.22–1.06	67.88
	Undefatted	Whole seed	2.77 ± 0.12 ^b,z^	4.55 ± 0.26 ^b,w^	3.32 ± 0.17 ^b,y^	3.90 ± 0.23 ^b,x^	2.77–4.55	17.36
		Seed coat	124.16 ± 6.61 ^a,x^	135.30 ± 0.57 ^a,w^	89.25 ± 0.81 ^a,z^	110.44 ± 2.07 ^a,y^	89.25–135.30	14.96
		Dehulled seed	0.46 ± 0.03 ^b,x^	1.07 ± 0.02 ^c,w^	0.26 ± 0.01 ^c,y^	0.20 ± 0.05 ^c,z^	0.20–1.07	69.54
TEAC (mg TE/g)	Defatted	Whole seed	13.72 ± 0.28 ^b,w^	12.44 ± 2.15 ^b,w^	7.21 ± 0.86 ^b,y^	9.83 ± 1.30 ^b,x^	7.21–13.72	23.18
		Seed coat	107.24 ± 2.52 ^a,wx^	93.46 ± 7.40 ^a,y^	114.91 ± 3.80 ^a,w^	99.33 ± 10.27 ^a,xy^	93.46–114.91	7.81
		Dehulled seed	2.36 ± 0.05 ^c,w^	2.51 ± 0.12 ^c,w^	1.97 ± 0.09 ^c,x^	2.08 ± 0.14 ^b,x^	1.97–2.51	9.60
	Undefatted	Whole seed	13.77 ± 0.56 ^b,w^	10.39 ± 2.52 ^b,x^	8.18 ± 0.44 ^b,z^	9.47 ± 1.53 ^b,xy^	8.18–13.77	19.78
		Seed coat	98.55 ± 2.07 ^a,x^	105.82 ± 7.35 ^a,wx^	108.81 ± 3.74 ^a,w^	99.29 ± 10.64 ^a,x^	98.55–108.81	4.21
		Dehulled seed	2.33 ± 0.04 ^c,x^	2.62 ± 0.05 ^c,w^	2.15 ± 0.01 ^c,y^	1.98 ± 0.04 ^b,z^	1.98–2.62	10.28

Different superscript letters (^a–c^) within a column of the same cultivar and extract denote statistically significant differences (*p* < 0.05). Different superscript letters (^x,y,w,z^) within a row and the same seed part denote statistically significant differences (*p* < 0.05). DPPH: DPPH-radical scavenging activity; FRAP: Ferric reducing antioxidant power; TEAC: Trolox equivalent antioxidant activity.

**Table 4:** Analysis of variance showing the effects of cultivar, extract type, seed parts and their interactions on metabolite contents and antioxidant activities in adzuki beans.

Source of Variation	Df	SS	*F*-Value
Total phenolic content			
Cultivar (C)	3	324.97	156.35 ****
Extract (E)	1	1.44	2.08
Seed part (S)	2	36,612.03	26,421.48 ****
C × E	3	2.11	1.02
C × S	6	579.20	139.33 ****
E × S	2	0.41	0.30
C × E × S	6	7.35	1.77
Total saponin content			
Cultivar	3	75,940.45	15.48 ****
Extract	1	10,229.88	6.25 *
Seed part	2	8,437,242.12	2579.05 ****
C × E	3	3984.82	0.81
C × S	6	114,827.42	11.70 ****
E × S	2	17,748.72	5.43 **
C × E × S	6	6425.22	0.66
DPPH			
Cultivar	3	2350.67	3100.43 ****
Extract	1	0.18	0.72
Seed part	2	42,244.48	83,578.14 ****
C × E	3	0.47	0.63
C × S	6	4767.76	3144.24 ****
E × S	2	0.58	1.15
C × E × S	6	2.13	1.40
TEAC			
Cultivar	3	141.74	4.15 *
Extract	1	1.70	0.15
Seed part	2	151,354.40	6641.79 ****
C × E	3	102.54	3.00
C × S	6	595.03	8.70 ****
E × S	2	1.31	0.06
C × E × S	6	300.85	4.40 ***
FRAP			
Cultivar	3	1974.74	174.77 ****
Extract	1	26.16	6.95 *
Seed part	2	196,956.24	26,146.63 ****
C × E	3	47.07	4.17*
C × S	6	3422.82	151.46 ****
E × S	2	52.10	6.92 **
C × E × S	6	106.69	4.72 ***

C: Cultivar; Df: Degree of freedom; DPPH: DPPH-radical scavenging activity; E: Extract; FRAP: Ferric reducing antioxidant power; S: Seed part; SS: Sum of squares; TEAC: Trolox equivalent antioxidant activity. * Significant at *p* ≤ 0.05; ** Significant at *p* ≤ 0.01; *** Significant at *p* ≤ 0.001; **** Significant at *p* ≤ 0.0001.

## Data Availability

Data are contained within the article or Supplementary Materials.

## Supplementary Materials

The following supporting information can be downloaded at: https://www.mdpi.com/article/10.3390/antiox11061134/s1, Table S1. Anthocyanin contents in the seed coats of four adzuki bean cultivars.
