# Supplementary material for: Variability of Anthocyanin Concentrations, Total Metabolite Contents and Antioxidant Activities in Adzuki Bean Cultivars
Source: Antioxidants (Basel). 2022 Jun 9;11(6):1134. doi: 10.3390/antiox11061134 (PMC9220110; doi:10.3390/antiox11061134)
Supplement: Supplementary file 1 [file antioxidants-11-01134-s001.zip › antioxidants-1762281-supplementary.pdf]

Variability of Anthocyanin Concentrations, Metabolite Contents and Antioxidant Activities in Adzuki bean Cultivars

Kebede Taye Desta, Hyemyeong Yoon, Myoung-Jae Shin, Sukyeung Lee, Xiao-Han Wang, Yu-Mi Choi * and Jung-Yoon Yi *

National Agrobiodiversity Center, National Institute of Agricultural Sciences, Rural Development Administration, Jeonju 54874, Korea; kehasiet20@rda.go.kr (K.T.D); hyemyeung1@rda.go.kr (H.Y.); smj1204@rda.go.kr (M.-J.S.); reset00@korea.kr; wang0530@rda.go.kr (X.-H.W.)

* Correspondence: ymchoi@rda.go.kr (Y.-M.C.); naaeskr@korea.kr (J.-Y.Y.)

Supplementary Materials

Table S1. Anthocyanin contents in the seed coats of four adzuki bean cultivars.

Compound	Black seed coat cultivars		Red seed coat cultivars	
	Chilbopat	Geomguseul	Arari	Hongeon
D-3,5-*O*-di-G (mg/g)	3.89 ± 0.25[b,B]	5.21 ± 0.43[b,A]	nd	nd
D-3-*O*-Ga (mg/g)	3.97 ± 0.14[b,B]	5.30 ± 0.44[b,A]	nd	nd
D-3-*O*-G (mg/g)	10.88 ± 0.50[a,A]	12.46 ± 0.64[a,A]	nd	nd
Pt-3-*O*-Ga (mg/g)	1.47 ± 0.18[c]	nd	nd	nd
D-3-*O*-Ru (mg/g)	1.19 ± 0.06[c,B]	1.44 ± 0.07[d,A]	nd	nd
C-3-*O*-G (mg/g)	0.87 ± 0.02[c,A]	0.86 ± 0.01[d,A]	nd	nd
Pt-3-*O*-G (mg/g)	3.29 ± 0.57[b,A]	3.41 ± 0.61[c,A]	nd	nd
TAC (mg/g)	29.51 ± 4.14 [A]	28.68 ± 1.67 [A]	-	-

Different superscript letters (a-d) in a column show significant differences between anthocyanins in a cultivar ($p < 0.05$).
Different superscript letters (A,B) in a row show significant differences of a target anthocyanin between cultivars ($p < 0.05$).
C-3-*O*-G: cyanidin-3-*O*-glucoside; D-3,5-*O*-di-G: delphinidin-3,5-*O*-diglucoside; D-3-*O*-Ga: delphinidin-3-*O*-galactoside; D-3-*O*-G: delphinidin-3-*O*-glucoside; D-3-*O*-Ru: delphinidin-3-*O*-rutinoside; Pt-3-*O*-Ga: petunidin-3-*O*-galactoside; Pt-3-*O*-G: petunidin-3-*O*-glucoside; TAC: total anthocyanin content. [nd]Not detected.
